# The role of orphanin FQ/nociceptin in neuroplasticity: relationship to stress, anxiety and neuroinflammation

**DOI:** 10.3389/fncel.2013.00173

**Published:** 2013-10-08

**Authors:** Elyse M. Mallimo, Alexander W. Kusnecov

**Affiliations:** ^1^Behavioral and Systems Neuroscience Program, Department of Psychology, Rutgers UniversityNew Brunswick, NJ, USA; ^2^Joint Graduate Program in Toxicology, Rutgers UniversityNew Brunswick, NJ, USA

**Keywords:** orphanin FQ, nociceptin, HPA axis, fear, astrocytes, microglia, cytokines, immune system

## Abstract

The neuropeptide, orphanin FQ/nociceptin (OFQ/N or simply, nociceptin), is expressed in both neuronal and non-neuronal tissue, including the immune system. In the brain, OFQ/N has been investigated in relation to stress, anxiety, learning and memory, and addiction. More recently, it has also been found that OFQ/N influences glial cell functions, including oligodendrocytes, astrocytes, and microglial cells. However, this latter research is relatively small, but potentially important, when observations regarding the relationship of OFQ/N to stress and emotional functions is taken into consideration and integrated with the growing evidence for its involvement in cells that mediate inflammatory events. This review will first provide an overview and understanding of how OFQ/N has been implicated in the HPA axis response to stress, followed by an understanding of its influence on natural and learned anxiety-like behavior. What emerges from an examination of the literature is a neuropeptide that appears to counteract anxiogenic influences, but paradoxically, without attenuating HPA axis responses generated in response to stress. Studies utilized both central administration of OFQ/N, which was shown to activate the HPA axis, as well as antagonism of NOP-R, the OFQ/N receptor. In contrast, antagonist or transgenic OFQ/N or NOP-R knockout studies, showed augmentation of HPA axis responses to stress, suggesting that OFQ/N may be needed to control the magnitude of the HPA axis response to stress. Investigations of behavior in standard exploratory tests of anxiogenic behavior (eg., elevated plus maze) or learned fear responses have suggested that OFQ/N is needed to attenuate fear or anxiety-like behavior. However, some discrepant observations, in particular, those that involve appetitive behaviors, suggest a failure of NOP-R deletion to increase anxiety. However, it is also suggested that OFQ/N may operate in an anxiolytic manner when initial anxiogenic triggers (eg., the neuropeptide CRH) are initiated. Finally, the regulatory functions of OFQ/N in relation to emotion-related behaviors may serve to counteract potential neuroinflammatory events in the brain. This appears to be evident within the glial cell environment of the brain, since OFQ/N has been shown to reduce the production of proinflammatory cellular and cytokine events. Given that both OFQ/N and glial cells are activated in response to stress, it is possible that there is a possible convergence of these two systems that has important repercussions for behavior and neuroplasticity.

## INTRODUCTION

Orphanin FQ/nociceptin (OFQ/N) is a heptadecapeptide most closely related to the endogenous kappa opioid peptide (KOP) dynorphin A (Meunier et al., 1995; Reinscheid et al., 1995). The peptide is referred interchangeably to either OFQ/N or nociceptin, due to its known hyperalgesic effects (Reinscheid et al., 1995). Early studies surrounding the isolation of this opioid-like neuropeptide revealed that OFQ/N lacks affinity for traditional opioid receptors (Bunzow et al., 1994; Mollereau et al., 1994; Reinscheid et al., 1996). Eventually it was shown that OFQ/N binds selectively to a G protein coupled receptor, the nociceptin opioid-like peptide (NOP) receptor (also known as ORL-1 or opioid-like orphan receptor 1), which resembles the classical opioid receptors in structure but does not bind opioid ligands (Bunzow et al., 1994; Fukuda et al., 1994; Mollereau et al., 1994; Wick et al., 1994). At the cellular level, activation of the NOP receptor (NOP-R) exerts inhibitory effects as it is negatively coupled to adenylyl cyclase (Mollereau et al., 1994; Wang et al., 1994), stimulates K^+^ conductance (Hawes et al., 2000) and inhibits high-voltage activated (HVA) N-type Ca^2+^ channels (Altier and Zamponi, 2008). As a result, OFQ/N binding to NOP-R reduces neuronal excitability and inhibits neurotransmitter release (Schlicker and Morari, 2000).

**FIGURE 1:**
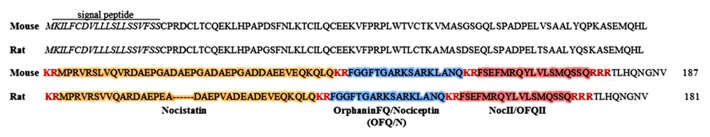
**Amino acid sequences for mouse (187 aa) and rat (181 aa) nociceptin precursors (ppOFQ/N).** The signal peptide is labeled and shown in italics. Proteolytic cleavage sites are shown in red, bold font. Enzymatic cleavage may occur at multiple sites along the ppOFQ/N peptide generating three biologically active neuropeptides; OrphaninFQ/Nociceptin (blue), Nocistatin (orange) and NocII/OFQII (red; Mollereau et al., 1996).

The OFQ/N peptide is derived through enzymatic cleavage from a larger precursor peptide, that also liberates other biologically active peptides (see **Figure 1**; Meunier et al., 1995; Okuda-Ashitaka et al., 1998; Reinscheid et al., 2000; Mogil and Pasternak, 2001). In this way, the nociceptin precursor, preproOFQ/N (ppOFQ/N), is similar to proopiomelanocortin (POMC), the precursor to several unrelated bioactive peptides (Meunier et al., 1995). More specifically, ppOFQ/N codes for two additional neuropeptides, nocistatin and NocII/OFQII, that lie immediately upstream and downstream, respectively, of OFQ/N (Meunier et al., 1995; Okuda-Ashitaka et al., 1998; Reinscheid et al., 2000). Less is known regarding the functional role of these neuropeptides, although there is evidence to suggest that nocistatin exerts biological effects opposite to those of OFQ/N (Okuda-Ashitaka et al., 1998; Gavioli et al., 2002).

High sequence homology between OFQ/N, NOP-R and components of the opioid superfamily prompted initial investigations into the role of OFQ/N in nociception (Meunier et al., 1995; Reinscheid et al., 1995; Rossi et al., 1998). Early findings revealed the relationship between nociceptin signaling and pain transmission to be complex as OFQ/N produced hyperalgesia when administered supraspinally (Mollereau et al., 1994; Reinscheid et al., 1995) and analgesia when administered at spinal sites (Rossi et al., 1998). Further investigation of the OFQ/N system revealed that both OFQ/N and its receptor are prominently expressed in stress-sensitive limbic and limbic-associated brain structures involved in processing emotionally relevant stimuli (Bunzow et al., 1994; Nothacker et al., 1996; Neal et al., 1999a, b; Pampusch et al., 2000). Since this suggested a potential role for OFQ/N in behaviors other than nociception, a good deal of effort has been directed to determining the role of OFQ/N in stress-related endocrine functions and behavior. Moreover, several reviews have highlighted the importance of OFQ/N in the modulation of reward, addiction and learning (Noda et al., 2000; Koob, 2008; Kuzmin et al., 2009; Zaveri, 2011).

In the current review, focus will be placed on the ability of OFQ/N to modulate anxiety-related behaviors and stress-induced neuroendocrine responses. In addition, the emerging role of OFQ/N in immune and/or inflammatory processes will be considered. There is considerable evidence that OFQ/N and NOP-R are expressed in endocrine (i.e., adrenal gland), and lymphoid organs and cells (e.g., blood leukocytes), including those considered to constitute “immune-like” functions in the CNS (Wang et al., 1994; Halford et al., 1995; Lachowicz et al., 1995; Nothacker et al., 1996; Peluso et al., 1998; Pampusch et al., 2000; Finley et al., 2008). Importantly, broad yet restricted expression of OFQ/N and NOP-R throughout the CNS and periphery suggests a modulatory role for the nociceptin system in regulation of emotional states, cognitive processes, and neuroendocrine and immune functions (Halford et al., 1995; Mamiya et al., 1998; Nabeshima et al., 1999; Devine et al., 2001; Leggett et al., 2009a). Accordingly, this paper will address the large but controversial literature concerning the role of OFQ/N in modulating physiological and behavioral responses to stress. In addition, the putative role of the nociceptin system in regulating neuroinflammatory processes will be discussed. This research is potentially important, given the relationship of OFQ/N to stress and emotional functions, and the increasing relevance of this to inflammatory events.

## ORPHANIN FQ/NOCICEPTIN AND RESPONSES TO PSYCHOGENIC STRESS

Stress as a physiological state originates from both systemic and psychogenic origins impacting nervous, endocrine and immune system functions. Of particular interest is the hypothalamic-pituitary-adrenal (HPA) axis, a major branch of the neuroendocrine system and the principle endocrine component of the stress response. Exposure to stress, both physical and psychological, activates the HPA axis, triggering a neuroendocrine cascade that culminates in the release of glucocorticoids [cortisol in humans and corticosterone (CORT) in rodents]. Briefly, stressor exposure activates neurosecretory cells of the paraventricular nucleus (PVN) of the hypothalamus, which secrete corticotropin-releasing hormone (CRH; also known as CRF) into the hypophyseal portal circulation. Once in the anterior pituitary, CRH stimulates CRH receptor I (CRH-RI) and induces the release of adrenocorticotropic hormone (ACTH), which is released into the systemic circulation, and subsequently stimulates the secretion of glucocorticoids from the adrenal cortex. A variety of stimuli can serve as stressors to the activation of the HPA axis, including psychogenic, physical and immunologic stressors. In most cases, such stressors engage higher level neural processes mediated by limbic forebrain structures, such as the amygdala, bed nucleus of the stria terminalis (BNST) and medial prefrontal cortex (mPFC), all of which either directly or indirectly form connections that ultimately impact the PVN (Ulrich-Lai and Herman, 2009).

The stress-induced elevation of CORT serves to mobilize energy stores and increase arousal. In this way, glucocorticoids are beneficial for short-term survival as they prepare animals to cope with real or perceived threats to homeostasis. However, prolonged exposure to glucocorticoids can, among other things, suppress normal immune function and impair cognition. For this reason, glucocorticoid release following stressor exposure is tightly regulated and involves integration of complex feed-forward and feedback signals at the level of the brain. Notably, circulating glucocorticoids provide feedback inhibition that is mediated, in part, by limbic-associated structures (Jankord and Herman, 2008; Ulrich-Lai and Herman, 2009). Specifically, while there are multiple levels of glucocorticoid feedback, binding of CORT to glucocorticoid receptors (GR) in the hippocampus and mPFC contributes to the inhibitory regulation of HPA axis activation (Herman et al., 2012).

**FIGURE 2:**
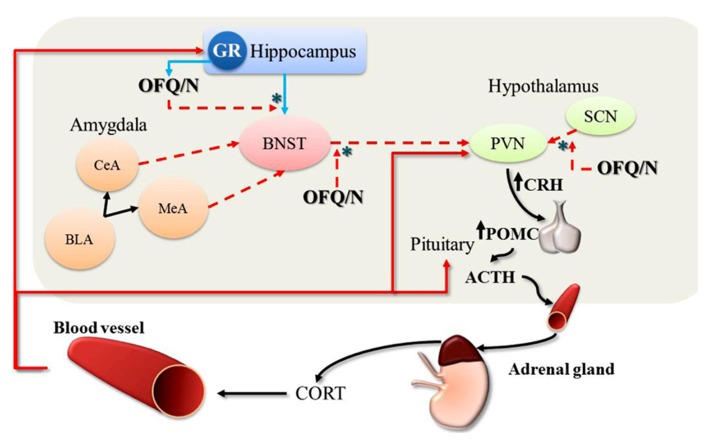
**Feed-forward effects of OFQ/N on the hypothalamic-pituitary-adrenal (HPA) axis response to stress**. The stimulatory effects of OFQ/N on the HPA axis are mediated through disinhibitory signaling (*) in multiple brain regions. Following activation of the HPA axis, CORT is released into circulation. At the level of the hippocampus, CORT binds to and stimulates GR which provides negative feedback control over HPA axis activity (red solid arrows). Binding of CORT to GR also induces the expression of OFQ/N, which binds to NOPr in the hippocampus and inhibits glutamatergic transmission (blue solid arrows) thereby removing trans-synaptic inhibition of the PVN. Nociceptin signaling also inhibits GABAergic outputs (red dashed arrows) from the BNST to the PVN, which attenuates the restraining influence of the BNST on PVN activity. Finally, nociceptin may also exert feed-forward effects on the HPA axis by suppressing inhibitory output from the SCN to the PVN. In this way, OFQ/N stimulates PVN neurons indirectly to produce CRH, which induces the expression of POMC and stimulates the release of ACTH. Collectively, these changes prolong or enhance the HPA axis response to stress. [Note: although depicted in this schematic for completeness, OFQ/N does *not* modulate amygdalar-outputs (CeA, central amygdala; MeA, medial amygdala; BLA, basolateral amygdala) to the PVN].

Accumulating evidence strongly implicates the nociceptin system in regulation of the stress response in general, and emerging data suggest that OFQ/N may contribute to feed-forward regulation of the HPA axis specifically. Therefore, evidence is reviewed regarding the involvement of endogenous OFQ/N in the regulation of the HPA axis response to acute psychogenic stress. A summary of these findings is provided in **Tables 1** and **2**, as well as **Figure 2**.

**Table 1:** Effects of manipulating the nociceptin system on the hypothalamic-pituitary-adrenal (HPA) axis response in stressed and non-stressed animals.

OFQ/N manipulation under resting conditions			Neuroendocrine effect	
Manipulation	Species	Dose (route of administration)	Hypothalamus	Pituitary	Plasma	Reference
OFQ/N infusion	Rats	0.10–1.0 nmole (icv)	N/A	N/A	↑ ACTH	Devine et al. (2001)
					↑ CORT
	Rats	2.55 nmole (icv)	N/A	N/A	↑ ACTH	Nicholson et al. (2002)
	Rats	0.10–10 μg/rat (icv)	↑ CRH mRNA (PVN)	↑ POMC mRNA	↑ ACTH	Leggett et al. (2006)
					↑ CORT
	Rats	3 nmole (icv)	↑ # of c-Fos+ cells (PVN, SON)	N/A	N/A	Olszewski et al. (2000)
JTC-801	Rats	0.05 mg/kg (iv)	N/A	N/A	↑ CORT	Delaney et al. (2012)
		1 μg/rat (icv)	↑ # of c-Fos+ cells (PVN)	N/A	N/A
OFQ/N^-/-^ knockout	Mice	N/A	N/A	N/A	↑ CORT (basal vs OFQ/N+/+)	Koster et al. (1999)

**OFQ/N manipulation during stressor exposure**		
**Manipulation**	**Species**	**Dose (route of administration)/Type of stress**	**Hypothalamus**	**Pituitary**	**Plasma**	**Author**

OFQ/N + psychogenic stress	Rats	1.0 nmole (icv)/open field test	N/A	N/A	↑↑ ACTH	Devine et al. (2001)
					→ CORT
	Rats	0.01–1.0 nmole (icv)/open field test	N/A	N/A	↑↑ CORT	Fernandez et al. (2004)
	Rats	0.01–1.0 nmole (icv)/open field test	N/A	N/A	↑↑ CORT	Green et al. (2007)
	Rats	0.5–1.5 nmole (icv)/elevated plus maze	N/A	N/A	↑↑ CORT	Vitale et al. (2006)
UFP-101 + psychogenic stress	Rats	1 μg/rat (icv)/Restraint	Ø CRH mRNA (PVN)	Ø POMC mRNA	↑ CORT	Leggett et al. (2007)
OFQ/N^-/-^ knockout + psychogenic stress	Mice	Exposure to Elevated plus maze	N/A	N/A	↑↑ CORT (vs OFQ/N^+/+^)	Koster et al. (1999)

*OFQ/N, orphaninFQ/nociception; NOP-R, nociceptin receptor; OFQ/N^-/-^, nociceptin deficient (knockout); OFQ/N^+/+^, nociceptin intact (wildtype); JTC-801, NOP-R antagonist; UFP-101, NOP-R antagonist; icv, intracerebroventricular; iv, intravenous; CRH, corticotropin releasing hormone; PVN, paraventricular nucleus; SON, supraoptic nucleus; POMC, proopiomelanocortin; ACTH, adrenocorticotropic hormone; CORT, corticosterone; N/A, not applicable or effects not addressed in cited study; Ø, no effect; ↑, increase above baseline/corresponding control; ↑↑, *enhanced* elevation above corresponding control; →, prolonged elevation of stress-induced response.*

**Table 2:** Putative neural substrates mediating the stimulatory effects of OFQ/N on the hypothalamic-pituitary-adrenal (HPA) axis.

Region	Species	Supporting evidence	Reference
*Hypothalamus*	Rats	OFQ/N (0.10–1 μg, icv) ↑ CRH mRNA expression (PVN)	Olszewski et al. (2000);
		OFQ/N (3 nmole, icv) ↑ c-Fosimmunoreactivity (PVN)	Leggett et al. (2006)
*Pituitary*	Rats	OFQ/N (0.10–10 μg, icv) ↑ POMC mRNA	Leggett et al. (2006)
*Hippocampus*	Rats	Restraint stress ↑ OFQ/N release (CA1, CA3, DG)	Nativio et al. (2012)
*BNST*	Rats	Restraint stress ↓ OFQ/N content in forebrain region containing BNST (increased utilization?)	Devine et al. (2003)
	Rats	Intra-BNST injections (1.0 nmole) of OFQ/N ↑ circulating CORT	Green et al. (2007)
	Rats	OFQ/N (75 nM) inhibits neuronal firing in the BNST (*in vitro*)	Dawe et al. (2010)
*SCN*	Rats	OFQ/N (10–1000 nM) inhibited excitatory synaptic transmission in the SCN evoked by optic nerve stimulation (via presynaptic mechanisms)	Gompf et al. (2005)
		OFQ/N (3–1000 nM) inhibited GABA release from SCN neurons (via presynaptic mechanisms)
	Rats	OFQ/N (0.5 and 10 nM, icv) inhibited light-induced c-Fos expression in the SCN	Sugino et al. (2006)

*Abbreviations used (those not already indicated in **Table 1**): CA1, *cornuAmmonis* 1 region; CA3, *cornuAmmonis* 3 region; DG, dentate gyrus; BNST, bed nucleus of striaterminalis; SCN, suprachiasmatic nucleus; GABA, γ-aminobutyric acid; ↑, increased expression; ↓, decreased expression.*

## OFQ/N AND THE HYPOTHALAMIC-PITUITARY-ADRENAL AXIS

Studies concerning the role of the nociceptin system in regulation of the HPA axis consistently demonstrate that central administration of OFQ/N elevates circulating ACTH and plasma CORT in unstressed rats (Devine et al., 2001; Nicholson et al., 2002; Leggett et al., 2006). Moreover, OFQ/N enhances endocrine responses during neophobic tests of anxiety such as exposure to the open field (OF; Devine et al., 2001; Fernandez et al., 2004; Green et al., 2007) and elevated plus maze (EPM; Vitale et al., 2006). Such findings implicate OFQ/N in feed-forward regulation of the HPA axis, and further suggest that nociceptin signaling may mediate or contribute to the HPA axis response to psychogenic stress.

In apparent contrast to these findings, mice lacking the nociceptin gene (OFQ/N^-/-^) exhibit elevated basal and post-stress levels of plasma CORT relative to wildtype mice, suggesting that OFQ/N serves to dampen HPA axis output (Koster et al., 1999). While seemingly at odds with one another, the discrepant findings of Koster et al. (1999) and others (Devine et al., 2001; Nicholson et al., 2002; Fernandez et al., 2004; Leggett et al., 2006; Vitale et al., 2006; Green et al., 2007) could be ascribed to several factors. First, enhanced HPA axis activity in OFQ/N^-/-^ mice may be due to deficient OFQ/N signaling during key developmental processes rather than due to a lack of OFQ/N *per se*. In fact, ppOFQ/N and NOP-R mRNA transcripts are highly expressed during early, embryonic development, raising the possibility that OFQ/N signaling contributes to normal neuronal development (Ikeda et al., 1998). Of additional concern is that the ppOFQ/N gene, that was disrupted by Koster et al. (1999), encodes additional peptides that exert biological effects opposite to those of OFQ/N (Okuda-Ashitaka et al., 1998). Accordingly, the findings of Koster et al. (1999) might not be directly attributable to a loss of OFQ/N-NOP-R signaling.

These concerns notwithstanding, pharmacological blockade of NOP-R was shown to *enhance* stress-induced activation of the HPA axis (Leggett et al., 2007). Specifically, intracerebroventricular (icv) administration of the peptide NOP-R antagonist, UFP-101, enhanced and prolonged, respectively, ACTH and CORT responses to acute restraint (Leggett et al., 2007). This supports the notion that nociceptin signaling exerts a restraining effect on the HPA response to stressors (Koster et al., 1999), although under certain conditions UFP-101 acts as a partial agonist at the NOP-R receptor, potentially mimicking the effects of OFQ/N (Mahmoud et al., 2010). For example, under conditions in which NOP-R was highly expressed and constitutively active, UFP-101 inhibited Ca^2+^ currents in cultured, stellate ganglion neurons in a manner similar to that of OFQ/N (Mahmoud et al., 2010). On the other hand, UFP-101 blocked OFQ/N-mediated Ca^2+^ current inhibition in control cells expressing NOP-R at physiological levels (Mahmoud et al., 2010). Therefore, given its mixed pharmacological profile, it is uncertain whether in Leggett et al.’s (2007) study, UFP-101 enhanced restraint-stress induced activation of the HPA axis via partial agonist effects at NOP-R.

In a more recent study, the HPA modulatory effects of a different non-peptide NOP-R antagonist, JTC-801, were examined during exposure to an acute restraint stressor (Delaney et al., 2012). Although it did not further increase stress-induced levels of CORT, intravenous (iv) JTC-801 elevated plasma CORT in unstressed rats. This suggested that nociceptin tonically regulates, in an inhibitory fashion, basal activity of the HPA axis (Delaney et al., 2012), which contrasted with other evidence that UFP-101 did not alter basal HPA axis activity (Leggett et al., 2007). Once again, however, and like other NOP-R antagonists, JTC-801 exhibits a complex pharmacological profile including antagonist and inverse agonist actions (Mahmoud et al., 2010), and allosteric regulation (Sestili et al., 2004; Del Giudice et al., 2011). Therefore, other experimental factors could have influenced the effect of JTC-801 on the HPA axis including route of drug administration, dose-response characteristics, and the selectivity profile of JTC-801 at NOP-R (Delaney et al., 2012).

It seems, therefore, that pharmacological attempts to determine the role of OFQ/N in the HPA axis response to stress have been hampered by poorly selective NOP-R antagonists. Moreover, the lack of correspondence in methodology renders the results of these studies difficult to reconcile. Although this has created some uncertainty regarding whether OFQ/N is necessary for activation or inhibition of the HPA axis, it has not deterred efforts to identify the neural substrate(s) and signaling mechanism(s) that might underlie OFQ/N involvement in HPA axis regulation. One possibility is that nociceptin contributes to the synthesis and release of other major hormones or peptides that exert excitatory effects on the HPA axis. In fact, OFQ/N-induced activation of the HPA axis is paralleled by up-regulation of CRH mRNA in the PVN of the hypothalamus and increased expression of POMC mRNA in the pituitary (Leggett et al., 2006). Moreover, icv administration of OFQ/N reportedly increased Fos protein expression in the PVN (Olszewski et al., 2000). However, given that OFQ/N binding to NOP-R reduces neuronal excitability, inhibiting both glutamate and GABA release (Meis and Pape, 2001; Bianchi et al., 2004; Roberto and Siggins, 2006; Cruz et al., 2012), it is likely that activation of PVN neurons in response to OFQ/N occurs through indirect mechanisms.

It has been suggested that OFQ/N may drive HPA axis output by disrupting limbic feedback to the hypothalamus (Devine et al., 2001; Leggett et al., 2006; see **Figure 2**). It is recognized that the hippocampus expresses high levels of GR, and this serves as a major source of glucocorticoid-mediated feedback control over HPA axis output (Sapolsky et al., 1983). The hippocampal GR are bound by circulating CORT, one effect of which is enhancement of glutamatergic transmission (Jankord and Herman, 2008; Ulrich-Lai and Herman, 2009). This can result in trans-synaptic excitation of an inhibitory relay in the BNST that maintains and/or augments inhibition of the PVN (Jankord and Herman, 2008). This normal state of affairs, however, might be disrupted by OFQ/N (see **Figure 2**). Exposure of rats to acute restraint increases OFQ/N release in the hippocampus in an adrenal-dependent manner, and mimicked by CORT injection, suggesting that glucocorticoids are necessary for stress-induced hippocampal OFQ/N release (Nativio et al., 2012). However, it is not known whether this glucocorticoid-dependent hippocampal OFQ/N release subsequent to stressor exposure prolongs or enhances HPA axis activity.

Alternatively, there is evidence that OFQ/N release in the BNST may play a role in modulating the HPA axis. As mentioned, the neural circuitry for regulation of the HPA axis includes the BNST, which contains GABAergic cells that send afferents to the PVN and inhibit HPA responses to stress (Ulrich-Lai and Herman, 2009). Acute restraint stress was reported to reduce OFQ/N content in gross dissections of the basal forebrain region containing the BNST, and this was argued to reflect increased release and utilization of OFQ/N (Devine et al., 2003). Importantly, OFQ/N neurotransmission and stimulation of NOP-R in the BNST is thought to reduce GABAergic input to the PVN, which ultimately would prolong activation of the HPA axis and stress-induced elevations of CORT. Consistent with this hypothesis, OFQ/N elicits strong inhibition of BNST neuronal firing *in vitro* (Dawe et al., 2010). Moreover, unilateral, intra-BNST infusion of OFQ/N in rats dose-dependently enhanced the CORT response to mild psychogenic stress (exposure to an OF; Green et al., 2007). At the very least, therefore, it could be argued that OFQ/N-mediated signaling in the BNST has significant consequences on HPA axis functions precipitated by stressors; whether this also involves hippocampal recruitment as suggested above remains to be determined.

The amygdala has also received attention as a potential mediator of OFQ/N effects on the HPA axis (Green et al., 2007). A heterogeneous structure comprised of many nuclei, the amygdala is differentially recruited by systemic and psychogenic stressors, which affects the PVN indirectly through synaptic inputs in the BNST (Ulrich-Lai and Herman, 2009). The two major output nuclei of the amygdala (central and medial amygdala; CeA and MeA) contain inhibitory projection neurons that synapse onto GABAergic cell groups in the BNST (Ulrich-Lai and Herman, 2009). Therefore, by means of a disinhibitory process, activation of the CeA and/or MeA results in *excitation *rather than inhibition of the PVN. Interestingly, injections of OFQ/N into the amygdala did not alter the CORT response to a mild, psychogenic stressor (Green et al., 2007), in spite of other evidence that (1) social defeat stress induced NOP-R mRNA expression in the CeA and basomedial (BMA) nuclei of the amygdala (Green and Devine, 2009), (2) acute restraint stress reduced ppOFQ/N mRNA expression in the CeA (Delaney et al., 2012), and (3) icv administration of OFQ/N induced Fos protein expression in the CeA (Olszewski et al., 2000). Based on such evidence, it is not unlikely that OFQ/N signaling occurs in the amygdala, and can increase anxiety-like behavior (Green et al., 2007). However, such intra-amygdalar behavioral effects are likely to be dissociated from the neuroendocrine effects of OFQ/N, which most likely engage alternative pathways.

Finally, it has been suggested that the stimulatory effects of OFQ/N on glucocorticoid release arise from modulation of *circadian* inputs to the HPA axis (Devine et al., 2001; Leggett et al., 2007). In rodents, blood CORT concentrations are lowest during the light or diurnal phase of the circadian cycle, and peak in the nocturnal phase, when rodents display high levels of activity, including increased food and water intake. Similarly, CORT-mediated negative feedback regulation of ACTH varies across the circadian light/dark cycle. For example, exposure to stress during the nadir or low phase of the CORT circadian rhythm induces an ACTH response of greater magnitude than during the peak phase. A critical structure in the regulation of circadian rhythms is the suprachiasmatic nucleus (SCN) of the hypothalamus, which relays photic information from the retina to the PVN. Interestingly, NOP-R is prominently expressed in the SCN, and OFQ/N has been shown to alter the activity of SCN neurons both *in vitro* (Gompf et al., 2005) and *in vivo* (Sugino et al., 2006). For example, icv administration of OFQ/N suppressed light-induced c-Fos expression in the SCN of rats (Sugino et al., 2006), while OFQ/N inhibited both excitatory and inhibitory neurotransmission in SCN neurons (Gompf et al., 2005). In view of these findings, it has been hypothesized that OFQ/N might stimulate the HPA axis and enhance endocrine responses to acute stress by altering circadian input to the PVN across the light/dark cycle (Devine et al., 2001; Leggett et al., 2007). Although it is also likely that OFQ/N-dependent modulation of the HPA axis response to acute psychogenic stress occurs through additive actions in multiple limbic and extra-limbic sites (Devine et al., 2001; Green et al., 2007).

In summary, there is consistent evidence that OFQ/N is involved in the activation of the HPA axis. What is not clear at present is the precise mechanism by which this occurs. One factor that has not been fully investigated with regard to the HPA axis is the role of neurotransmitter systems regulated by OFQ/N. For example, there is evidence to suggest that OFQ/N exerts an inhibitory effect on the release of acetylcholine (ACh) in cortical, hippocampal and striatal brain regions (Itoh et al., 1999; Cavallini et al., 2003; Uezu et al., 2005). Cholinergic stimulatory effects on the HPA axis have long been recognized (Matta et al., 1998; Rhodes et al., 2001), and it might be expected that if OFQ/N exerts an inhibitory effect on ACh, this could be one mechanism by which an inhibition of the HPA axis is maintained. Indeed, the elevated and/or prolonged HPA axis responses observed in the absence of OFQ/N or loss of NOP-R signaling (Koster et al., 1999; Mallimo et al., 2010), fit this particular explanatory scheme. However, whether modulation of the cholinergic system is involved, remains to be determined.

## OFQ/N AND REGULATION OF ANXIETY STATES

Anxiety as a mental state is characterized by heightened arousal, hypervigilance and the expression of defensive behaviors (e.g., escape or avoidance). Anxiety-responses are evoked in anticipation of threat and are accompanied by autonomic and endocrine activation. Collectively, these physiological and behavioral reactions are thought to be adaptive and prepare an individual to cope with unexpected or unavoidable situations. Although there is no shortage of peptides implicated in anxiety-like behavior, converging lines of evidence from rodent studies strongly implicate the nociceptin system in regulation of anxiety states. Notably, OFQ/N and NOP-R agonists have been shown to attenuate the expression of anxiety-like behaviors and in a manner comparable to that of conventional anxiolytics including diazepam (Jenck et al., 1997, 2000) and alaprozam (Jenck et al., 2000).

The attenuation of anxiety-like behaviors by OFQ/N stands in contrast to other evidence already discussed that nociceptin promotes feed-forward regulation of the HPA axis, which is often associated with anxiety states. Moreover, stimulation of the nociceptin system has also been demonstrated to *enhance* – rather than attenuate – the expression of anxiety-like behavior under specific conditions (Fernandez et al., 2004; Green et al., 2007). Therefore, it would appear that the relationship between the emotional and endocrine effects of nociceptin is not straightforward, nor is it clear whether the OFQ/N system is anxiolytic or anxiogenic. In the remainder of this section, these studies will be reviewed more closely, and are summarized in **Tables 3** and **4**.

**Table 3:** Effects of manipulation of the nociceptin system on the expression of innate (a) and learned (b) anxiety-like behavior in rodents.

		Manipulation	Effects of OFQ/N or analog (dose)		
Behavioral test/reference	Species	(Route of administration)	Anxiogenic behavior	Non-specific behavioral responses	
**(a) Ethologically based tests**

*Light-dark (LD) box*			*Avoidance of lighted area*	*Locomotor activity*	*Motor coordination*
Jenck et al. (1997)	Mice	OFQ/N (icv)	↓ (0.3–1.0 nmole)	N/A	#↓ (1.0–3.0 nmole)
Fernandez et al. (2004)	Rats		↑ (0.01–1.0 nmoles)	Ø (0.1 nmoles)	N/A

*Elevated plus maze (EPM)*			*Avoidance of open arms*	*Locomotor activity*	*Motor coordination*
Jenck et al. (1997)	Rats	OFQ/N (icv)	↓ (0.03–0.3 nmole)	#↓ (0.3–1.0 nmole)	#↓ (1.0 nmole)
Fernandez et al. (2004)			↑ (0.01–1.0 nmole)	↓ (1.0 nmole)	N/A
Gavioli et al. (2002)	Mice		↓ (0.01–0.1 nmol)	Ø (0.01–0.1 nmol)	Ø (0.01–0.1 nmol)
Uchiyama et al. (2008)			↓ (0.1–0.32 nmol)	↓ (0.1–0.32 nmol)	N/A
Jenck et al. (2000)****	Rats	Ro64-6198 (ip)	↓ (1.0–3.2 mg/kg)	Ø (1.0–3.2 mg/kg)	Ø (1.0–3.2 mg/kg)
Gavioli et al. (2002)		Nocistatin (icv)	↑ (0.1–3.0 pmol)	Ø (0.1–3.0 pmol)	Ø (0.1–3.0 pmol)
Rodi et al. (2008)	Rats
CRH (1 μg/rat, icv)		OFQ/N (icv)	↓ (1.8 μg/rat)	N/A	N/A
		OFQ/N (intra BNST)	↓ (0.5 μg/rat)	N/A	N/A

*Hole-board test*			*Reduced exploration (head-dipping)*	*Locomotor activity*	*Motor coordination*
Kamei et al. (2004)	Mice	OFQ/N (icv)	↓ (0.01 nmol)	Ø (0.01 nmol)	N/A
Florin et al. (1996)			↓ (0.05 nmol)	N/A	N/A

*Novelty-induced hypophagia*			*Hypophagia (novel environment)*	*Hypophagia (home cage)*
Goeldner et al. (2012)	Mice	Ro64-6198 (ip)	↓ (0.3–1.0 mg/kg)	Ø (0.3–1.0 mg/kg)	

*Exposure to a novel environment/open field (OF)*			*Thigmotaxic behavior*	*Locomotor activity*	*Motor coordination*
Green et al. (2007)	Rats	OFQ/N (icv)	↑ (0.01–1.0 nmoles)	Ø (1.0 nmoles)	N/A
		OFQ/N (intra-amygdaloid)	*↑ (0.1–1.0 nmoles)	N/A	N/A
		OFQ/N (intra-BNST)	*↑ (1.0 nmoles)	N/A	N/A
Fernandez et al. (2004)		OFQ/N (icv)	↑ (0.001–1.0 nmoles)	N/A	N/A
Florin et al. (1996)	Mice	OFQ/N (icv)	N/A	↑ (0.005–5.0 nmol)	N/A
Jenck et al. (1997)			N/A	↑ (0.1 nmole)	N/A

*Urocortin (0.06 nmole, icv)-induced hypolocomotion*			*Hypolocomotion*		
Jenck et al. (1997)	Mice	OFQ/N (icv)	↓ (0.1–0.3 nmole + urocortin)	

*CRH-induced Hypophagia*			*Hypophagia*	*Hyperphagia (food-satiated or deprived rats)*	
Ciccocioppo et al. (2003)	Rats		
CRH (0.04 nmol, icv)		OFQ/N (intra-BNST)	↓ (0.02–0.21 nmol)	Ø (0.02–0.21 nmol)	
Ciccocioppo et al. (2004)	Rats		
CRH (0.2 μg/rat, icv)		OFQ/N (icv)	↓ (1 μg/rat)	Ø (1 μg/rat)	
			Ro64-6198 (ip)	↓ (0.3–1.0 mg/kg)	Ø (0.3–1.0 mg/kg)	
**(b) Models of learned anxiety or fear**
*Operant Conflict Procedure*			*Punished responding*	*Unpunished responding*	
Jenck et al. (1997)	Mice	OFQ/N (icv)	↑ (3 nmoles)	Ø (3 nmoles)	
Jenck et al. (2000)	Rats	Ro64-6198 (ip)	↑ (1.0 mg/kg)	Ø (1.0 mg/kg)	
Varty et al. (2005)			↑ (3.0–10.0 mg/kg)	Ø (3.0–10.0 mg/kg)	

*Fear potentiated startle (FPS)*			*Response amplitude (CS+)*	*Response amplitude (habituation trials)*	
Jenck et al. (2000)	Rats	Ro64-6198 (ip)	↓ (3.2–10 mg/kg)	Ø (3.2–10 mg/kg)	

*Elevated T-maze*			*Inhibitory avoidance behavior*	*Locomotor activity*	*Motor coordination*
Duzzioni et al. (2011)	Rats	UFP-101 (icv)	↓ (1.0–10.0 nmol)	*↑ or ↓ (dose dependent)	N/A

Abbreviations used (those not already defined in**Tables 1** and **2**): Ro64-6198, nociceptin receptor agonist;↓ decreased expression of behavior; ↑increased expression of behavior; Ø no effect on behavior; N/A, not applicable or effects not addressed in referenced study; # non-significant trend toward increased or decreased expression of behavior; *statistically significant effect for some but not all aspects of measured behavior; CS+, presence of conditioned stimulus. Note: effects of OFQ/N or analog on motor coordination are summarized for effective drug doses employed in other behavioral tests.

**Table 4:** Effects of targeted deletion of endogenous OFQ/N and NOP-R signaling on the expression of (a) innate and (b) learned anxiety-like or fear-like behavior in rodents.

			Effect of genetic manipulation		
Behavioral test/reference	Species	Transgenic Model	Anxiogenic behavior	Non-specific behavioral responses	
**(a) Ethologically based tests**

*Light-dark (LD) box*			*Avoidance of lit area*	*Locomotor activity*	*Motor coordination*
Koster et al. (1999)	Mice	OFQ/N^-/-^ knockout	↑ (vs OFQ/N^+’+^)	N/A	N/A
Gavioli et al. (2007)		NOP-R^-/-^knockout	↑ (vs NOP-R^+/+^)	↑ (vs NOP-R^+/+^)	N/A
Ouagazzal et al. (2003)		
Crowded housing (5/cage)			↑ (vs OFQ/N^+’+^)	N/A	N/A
†Single-housed			Ø	N/A	Ø

*Elevated plus maze (EPM)*			*Avoidance of open arms*	*Locomotor activity*	*Motor coordination*
Koster et al. (1999)	Mice	OFQ/N^-/-^ knockout	↑ (vs OFQ/N^+’+^)	Ø	N/A
Gavioli et al. (2007)		NOP-R^-/-^knockout	↑ (vs NOP-R^+/+^)	↑ (vs NOP-R^+/+^)	N/A
Mamiya et al. (1998)			Ø	Ø	Ø
Rizzi et al. (2011)	Rats		↑ (vs NOP-R^+/+^)	Ø	↑ (vs NOP-R^+/+^)

*Elevated T-maze*			*Escape behavior*		
Gavioli et al. (2007)	Mice	NOP-R^-/-^knockout	*↓ *(vs NOP-R^+/+^)	

*Hole-board test*			*Reduced exploration (head dipping)*	*Locomotor activity*	*Motor coordination*
Gavioli et al. (2007)	Mice	NOP-R^-/-^knockout	Ø	N/A	N/A

*Marble-burying test*			*Burying behavior*		
Gavioli et al. (2007)	Mice	NOP-R^-/-^knockout	Ø	

*Novelty-induced hypophagia*			*Hypophagia (novel environment)*	*Hypophagia (home cage)*	
Gavioli et al. (2007)	Mice	NOP-R^-/-^knockout	*↓ *(vs NOP-R^+/+^)	Ø

*Exposure to a novel environment/open field (OF)*			*Thigmotaxic behavior*	*Locomotor activity*	*Motor coordination*
Gavioli et al. (2007)	Mice	NOP-R^-/-^knockout	Ø	Ø	N/A
Koster et al. (1999)		OFQ/N^-/-^ knockout	↑ (vs OFQ/N^+’+^)	↓ (vs OFQ/N^+’+^)	N/A
Ouagazzal et al. (2003)			
†Single-housed			N/A	Ø	N/A
Rizzi et al. (2011)	Rats	NOP-R^-/-^knockout	Ø	Ø	↑ (vs NOP-R^+/+^)

			**Effect of genetic manipulation**

Behavioral test/reference	Species	Transgenic Model	Anxiogenic behavior
**(b) Models of learned anxiety or fear**
*Acoustic startle reflex procedure*			*Startle response amplitude*
Ouagazzal et al. (2003)			
Crowded housing (5/cage)	Mice	OFQ/N^-/-^ knockout	↑ (vs OFQ/N^+’+^)
†Single-housed			Ø

*Stress-induced analgesia (SIA)*			*SIA*
Koster et al. (1999)	Mice	OFQ/N^-/-^ knockout	Lack of habituation of SIA in response to repeated swim stress

*NOP-R^-/-^, nociceptin receptor deficient (knockout); NOP-R^+/+^, nociceptin receptor intact (wildtype); ↓ decreased expression of behavior; ↑ increased expression of behavior; Ø, no effect on behavior; N/A, not applicable or effects not addressed in referenced study; †, single-housed mice were maintained in adjacent, transparent cages that allowed for auditory, visual, and olfactory communication. This type of housing arrangement could have reduced the anxiogenic effects of isolation. Note: where applicable, effects of genotype on motor coordination (↑ enhanced; ↓ impaired) are summarized for each study.*

The modulatory effects of OFQ/N have been extensively studied in a variety of tests that model different aspects of anxiety in rodents. For instance, in mice and rats central administration of OFQ/N increased the amount of time spent in the lighted compartment of a light-dark (LD) box and enhanced open arm exploration in the EPM test (Jenck et al., 1997; Gavioli et al., 2002; Uchiyama et al., 2008). Likewise, systemic administration to rats of Ro64-6198, a nonpeptide NOP-R agonist, that easily penetrates the blood-brain barrier, attenuated open-arm avoidance in the EPM (Jenck et al., 2000) and, in mice, increased the consumption of a familiar and highly palatable, liquid diet in a novel environment (Goeldner et al., 2012). These studies used ethologically based tests, which involved measures of unconditioned responses (e.g., approach-avoidance behavior) and are based on a rodent’s inherent fear of unprotected, brightly lit and unfamiliar spaces. Consequently, it is possible that OFQ/N may be effective in reducing anxiety-like behavior under conditions in which an anxiogenic response is unlearned or hard-wired.

One caveat, however, is that unconditional responding is susceptible to drug effects on general activity levels (Rodgers, 1997). Notably, the aforementioned studies used effective dose ranges at which OFQ/N has been reported to produce either hyper- or hypolocomotion in mice and rats (Reinscheid et al., 1995; Devine et al., 1996; Florin et al., 1996; Rizzi et al., 2001). Moreover, at higher doses, OFQ/N can even impair motor coordination and balance (Jenck et al., 1997, 2000). Indeed, dose-dependent effects of OFQ/N on locomotor activity are most apparent in the EPM. For instance, in mice,low doses of OFQ/N (0.01 nmol, icv) attenuated open arm avoidance without affecting other measures of locomotor activity (Gavioli et al., 2002), while higher doses of OFQ/N (0.1–0.3 nmol, icv) decreased open arm avoidance and also increased the total number of transitions between open and closed arms (Uchiyama et al., 2008). At high doses, therefore, the behavioral effects of OFQ/N may not be specific to the anxiolytic properties of the neuropeptide.

Similar concerns for the behavioral effects of OFQ/N were observed in the hole board and LD emergence tests (Florin et al., 1996; Jenck et al., 1997; Kamei et al., 2004). In the hole board test, OFQ/N was thought to attenuate anxiety by virtue of increasing head-dipping behavior (into the holes of the apparatus), and this occurred without changing general activity level (Kamei et al., 2004). Moreover, this occurred at the lowest dose tested (0.01 nmol, icv; Kamei et al., 2004). However, in other studies the increased exploration (i.e., head dipping) in the hole board test, as well as emergence into light in the LD box test, could not be differentiated from the locomotor-stimulating properties of the OFQ/N dose range tested (0.05–1.0 nmol, icv; Florin et al., 1996).

One further consideration is that the specific parameters of locomotion may inform interpretation of the locomotor effects of a given treatment. For instance, although OFQ/N (0.05–0.5 nmol, icv) produced a general hyperlocomotion in mice subjected to an unfamiliar or novel environment (Florin et al., 1996; Jenck et al., 1997), this was parsed into increased horizontal movement and increased vertical movement (e.g., rearings). Such a combination of movements might be considered an index of increased exploration and possibly reduced anxiety in animals. Indeed, when *hypo*locomotion was induced by the CRH receptor II agonist urocortin (a purported anxiogenic effect), OFQ/N (0.1–0.3 nmol, icv) reversed this effect, and in a manner comparable to that of diazepam (Jenck et al., 1997). This may support the concept introduced above with regard to ethological tests: that OFQ/N is anxiolytic during states of heighted anxiety, serving to interfere with other neurochemical systems that initially trigger the anxiety-like behavior.

The anxiolytic properties of OFQ/N signaling have also been demonstrated in models of learned anxiety and/or fear (Jenck et al., 1997, 2000; Varty et al., 2005). In one study, OFQ/N attenuated response inhibition in a modified Geller-Seifter conflict test (Jenck et al., 1997). In this test, food-deprived mice are first trained to lever-press for food reinforcement. After training, mice are subjected to alternating non-conflict and conflict sessions, in which bar presses are either reinforced with food (non-conflict) or result in food delivery and simultaneous foot-shock (conflict). In vehicle-treated mice, foot-shock resulted in a selective inhibition of lever-press behavior. Conversely, OFQ/N increased operant responding during conflict sessions (Jenck et al., 1997), as did Ro64-6198 in rats (Jenck et al., 2000; Varty et al., 2005). This suggests that OFQ/N may reduce the degree of task-specific anxiety associated with punished responding.

It should be noted that OFQ/N is a potent orexigenic agent (Pomonis et al., 1996; Stratford et al., 1997; Polidori et al., 2000). In fact, administration of OFQ/N into the lateral or third ventricles stimulates feeding in non-food deprived rats (Pomonis et al., 1996; Polidori et al., 2000). Moreover, site-specific microinjection of OFQ/N into brain regions implicated in the regulation of food intake (Stratford et al., 1997; Polidori et al., 2000) induces hyperphagia in free-feeding rats. Therefore, in tasks that involve appetitive behavior, it is possible that the hyperphagic effects of nociceptin contribute to changes in behavioral responses observed in food-deprived animals, such as those trained in the Geller-Seifter conflict test. However, since neither OFQ/N nor Ro64-6198 affected lever press behavior during non-conflict sessions (i.e., unpunished responding), it is likely that the OFQ/N-induced increase in punished responding is specific to the anxiolytic properties of OFQ/N (Jenck et al., 1997, 2000; Varty et al., 2005).

Finally, OFQ/N signaling attenuates anxiogenic behavior in other models of conditioned or learned emotional responses, such as the fear-potentiated startle procedure (Jenck et al., 2000). In this paradigm a previously neutral, conditional stimulus (CS; e.g., a light) is explicitly paired with the presentation of an aversive, unconditional stimulus (US; e.g., foot-shock). Following repeated CS-US presentations, an association between the two stimuli is formed such that the light comes to predict the occurrence of foot-shock. After conditioning, the startle response to an acoustic stimulus (e.g., a short burst of white noise) is measured in the absence (CS-) and presence (CS+) of the CS on alternating trials. The magnitude of startle potentiation, or increase in response amplitude on trials in which the CS is present (CS+ trials), relative to trials in which the CS is absent (CS-), is then taken as an index of learned fear. Thus, animals should exhibit a greater startle response on trials in which the CS is presented in conjunction with the acoustic stimulus. Consistent with the anxiolytic-like effects of OFQ/N and Ro64-6198 in the Geller-Seifter conflict test (Jenck et al., 1997, 2000; Varty et al., 2005), systemic administration of Ro64-6198 attenuated the magnitude of startle potentiation in rats (Jenck et al., 2000). This reinforced the notion that fear and/or anxiety can be reduced after stimulation of the OFQ/N receptor NOP.

The use of transgenic mice deficient in various components of the OFQ/N system have also suggested that OFQ/N is involved in emotion regulation. For example, mutant mice lacking the OFQ/N gene (OFQ/N^-/-^) exhibited increased avoidance behavior in the EPM (Koster et al., 1999). More specifically, OFQ/N^-/-^ mice made fewer entries into and spent less time in the open arms of the EPM. Similarly, in the LD emergence test, OFQ/N^-/-^ mice, when compared to wildtype mice, spent less time exploring, and showed a higher latency to first enter, the lighted compartment. Mutant mice also showed a reduction in free exploration in the OF test, as evidenced by increased thigmotaxic behavior and hypolocomotion (Koster et al., 1999). Collectively, these findings provided converging evidence that endogenous OFQ/N signaling mediates behavioral responding in models of innate anxiety.

Interestingly, there is evidence that the behavioral phenotype of OFQ/N^-/-^ mice is influenced by social housing conditions (Koster et al., 1999; Ouagazzal et al., 2003). When maintained under crowded housing conditions (5 mice/cage), OFQ/N mutant mice displayed greater home-cage aggression and heightened emotional reactivity in unconditioned (LD box) and conditioned (acoustic startle) response tests. Interestingly, when mice were housed individually no behavioral differences were observed between the genotypes (Ouagazzal et al., 2003). Although isolation is also considered to be stressful the authors noted that single-housed mice were maintained in adjacent, transparent cages that allowed for auditory, visual, and olfactory communication. By providing some degree of interaction this type of housing arrangement could have reduced the anxiogenic effects of isolation. Nonetheless, these findings suggest that deficient OFQ/N signaling may confer a vulnerability to stress, and in this case, to the chronic stress produced by crowded housing conditions (Ouagazzal et al., 2003). More broadly, these data implicate endogenous OFQ/N in stressor-induced behavioral adaptation (Ouagazzal et al., 2003).

In good agreement with this idea, Koster et al. (1999) disrupted the ppOFQ/N gene, and reported that group-housed OFQ/N^-/-^ mice failed to adapt to repeated stressor exposure. Specifically, mice were subjected to a forced swim stressor, and adaptation to repeated swim was assessed by habituation of stress-induced analgesia (SIA). In contrast to wildtype mice, OFQ/N^-/-^ mice failed to adapt to repeated forced swim exposure and continued to exhibit SIA after each trial (Koster et al., 1999). These findings suggest that OFQ/N may be needed to promote behavioral resilience and maintenance of normal physiological functions during periods of repeated or chronic stress.

Though evidence from knockout studies corroborates the idea that OFQ/N likely serves to dampen the expression of anxiogenic-like behavior, it should be noted that the aforementioned use of OFQ/N^-/-^ mice involved a disruption in the ppOFQ/N gene. As noted earlier, this precursor peptide is cleaved to form other biologically active peptides (Meunier et al., 1995; Okuda-Ashitaka et al., 1998; Reinscheid et al., 2000; Mogil and Pasternak, 2001), such as nocistatin, which can have opposite biological effects to that of OFQ/N (Okuda-Ashitaka et al., 1998; Gavioli et al., 2002). For example, central administration of nocistatin alone *increased* anxiogenic behavior in rats tested in the EPM, and furthermore, abrogated the anxiolytic-like effects of OFQ/N in this same test (Gavioli et al., 2002). Therefore, behavioral data from mice with disruptions to ppOFQ/N must be carefully interpreted.

Nonetheless, it is encouraging that targeted deletions of NOP-R can produce a similar anxiogenic profile in both mice (Gavioli et al., 2007) and rats (Rizzi et al., 2011). For instance, NOP-R knockout animals (NOP-R^-/-^) spend less time in the ostensibly anxiety-provoking open areas of the EPM (Gavioli et al., 2007; Rizzi et al., 2011) and the illuminated region of the LD box (Gavioli et al., 2007). However, this not a consistent observation (Mamiya et al., 1998; Gavioli et al., 2007). Whereas Gavioli et al. (2007) describe an anxiogenic-like profile for NOP-R^-/-^ mice in the EPM, Mamiya et al. (1998) observed no behavioral differences between the wildtype and mutant genotypes also tested in the EPM. While not easily explained, it should be noted, that the genetic background on which the NOP-R mutation was generated varied between the two studies. As this is a critical factor in behavioral phenotypes of genetically modified mice, particularly with respect to anxiogenic behavior (Anisman et al., 2008), differences in background strain could have accounted for the disparate findings. For example, attending only to the data from wildtype mice, baseline levels of anxiety differed significantly between the studies (Mamiya et al., 1998; Gavioli et al., 2007), attesting to the importance of background genetics modifying the expression of gene deletions (Anisman et al., 2008).

Leaving aside the issue of background genetics in the interpretation of specific gene deletions, it should be emphasized that variations in the type of behavioral test can also reveal different results. For instance, Gavioli et al. (2007) report no behavioral differences between NOP-R^+/+^ and NOP-R^-/-^ mice in the OF, hole board and marble burying tests. These findings stand in contrast to other evidence that pharmacological stimulation of NOP-R attenuates anxiogenic-related behaviors in the OF and hole board tests (Florin et al., 1996; Jenck et al., 1997, 2000; Kamei et al., 2004). However, a variety of factors could have accounted for the discrepant findings. As noted by Gavioli et al. (2007), differences in the experimental conditions between their study and others (Florin et al., 1996; Jenck et al., 1997, 2000; Kamei et al., 2004) could have limited their ability to detect differences in anxiogenic-like behavior between the genotypes. It is also possible that other biological mechanisms compensated for the loss of NOP-R signaling throughout development and that these compensatory systems were sufficient to support normal, behavioral responding under certain experimental conditions.

Adding to the confusion emerging from transgenic mouse studies, NOP-R^-/-^ mice exhibited an *anxiolytic-like* profile when tested in the novelty-induced suppressed feeding behavior test (Gavioli et al., 2007). Briefly, in this test food-deprived mice are placed into the corner of an OF that, prior to testing, was baited with food pellets located in the centermost region of the OF. During testing, the latency to venture out and eat, and the amount consumed (over a 5 min period), was then scored for each animal. Immediately thereafter, mice were returned to their home cages where they were scored using the same behavioral parameters. Anxiety generated by the novel and ostensibly aversive environment (the OF) suppressed the motivation to seek and consume food, resulting in an increased latency to first eat, as well as a reduced food intake. However, upon returning to their home cages, animals quickly approached and consumed a greater amount of food than in the novel environment. Unexpectedly, NOP-R^-/-^ mice exhibited a reduced latency to eat and consumed a greater amount of food than wildtype controls in the OF (Gavioli et al., 2007). This apparent anxiolytic phenotype of NOP-R^-/-^ mice could not be accounted for by differences in appetite, as the body mass lost during deprivation was equivalent between the genotypes. Moreover, NOP-R^-/-^ and NOP-R^+/+^ mice consumed the same amount of food when returned to their home cages (Gavioli et al., 2007).

A different study by Mallimo et al. (2010) used ORL-1^-/-^ mice (i.e., NOP-R deficient) that received saline and immunologic treatment, and were tested within hours of treatment or several days later. Testing of mice included gustatory neophobia, OF behavior and exposure to a large and unfamiliar, novel object (to test for object neophobia). Of relevance to the current discussion is the behavior of the saline injected (ip) mice. In these mice, it was observed that relative to saline-injected ORL-1^+/+^ mice, gustatory neophobia was attenuated in ORL-1^-/-^ mice, which is in keeping with the observations of Gavioli et al. (2007) discussed above. Furthermore, and again similar to Gavioli et al. (2007), OF behavior tested several days later was not different between the ORL-1^-/-^ and ORL-1^+/+^ mice. However, in ORL-1^-/-^ mice, approach and contact with a novel object placed into the OF, was significantly reduced, and time spent away from the object was significantly increased (Mallimo et al., 2010).

These observations suggest that the impact of OFQ/N receptor deletion produces a complicated phenotype that appears to depend on the type of testing conducted on the animals. In particular, the use of an appetitive paradigm, such as those described above, does not unmask an anxiety-like pattern of behavior in mice rendered NOP-R deficient. Furthermore, there are alternative interpretations of the data that could be posited. For instance, a shorter latency to enter the centermost region of an OF to consume food, may reflect impulsive behavior. In line with this idea, UFP-101, a NOP-R antagonist, reduced the latency to first exit the closed arm of an elevated T-maze (ETM) and enter into an open, unprotected arm (Duzzioni et al., 2011). Although a reduced latency could be considered an index of a diminished anxiety-like behavior, it could also be argued that rats with shorter exit latencies are more impulsive than animals demonstrating an appropriate, inhibitory avoidance response. Although speculative, this hypothesis could account for some of the inconsistencies observed in the behavioral phenotype of NOP-R^-/-^ mice. At the very least, it is apparent that endogenous NOP-R differentially modulates anxiogenic-like behavior across different tests of anxiety, even when the behavioral methods employed intend to model the same aspects of anxiety (e.g., innate anxiety).

## CONCLUDING POINTS

In general, most of the behavioral evidence suggests that under conditions of stress, exposure to novel contexts, and learned anxiety and/or fear responses, OFQ/N is recruited to modulate the intensity or severity of behavioral change. Conceptually, it is tempting to think that OFQ/N serves as an attenuating influence on anxiety, although additional research is required to confirm this hypothesis, since not all of the data gathered to date fit neatly into this interpretation (Fernandez et al., 2004; Green et al., 2007). Still, it should be noted that the anxiogenic and anorectic effects of CRH are inhibited by OFQ/N (Ciccocioppo et al., 2003, 2004; Rodi et al., 2008), which reinforces the notion that OFQ/N functions as an opposing influence on neurochemical systems activated by stress. However, the precise effects of such opposition needs to be clarified. Interestingly, a recent study found that individuals who suffer from PTSD, and carry a single-nucleotide polymorphism for the human opioid receptor-like 1 gene (*Oprl1*), showed greater functional connectivity between the amygdala and the insula during exposure to fearful faces (Andero et al., 2013). Although not a therapeutic study, this study does provide the opportunity to determine whether PTSD symptoms might be better managed by administering OFQ/N receptor agonists. However, further work is going to be needed, since in the same report, ORL-1 receptor stimulation in mice prevented the consolidation of fear memory after an aversive event (ibid). How to implement treatments like this therapeutically without affecting other memory processes necessary for optimal cognitive functioning will require further analysis.

## OFQ/N AND INFLAMMATION

It is well established that immune factors – primarily cytokines – can impact neurobiological functions under normal and clinical conditions (Dantzer et al., 2008). Cytokines are immune-derived intercellular messengers that are involved in systemic inflammation, and their identification in damaged brain tissue has focused attention on neuroinflammatory mechanisms of brain dysfunction, as well as their possible contributions to brain plasticity and repair. Brain cytokines originate from glial cells, such as astrocytes, oligodendrocytes, and in particular, microglia (Ransohoff and Perry, 2009; Graeber et al., 2011). The regulation of cytokine synthesis and release is carefully orchestrated, since changes in the magnitude and duration of cytokine levels may modify a range of cognitive and emotional functions, including those aligned with classical indices of depressive symptomatology. Indeed, studies of immune challenge (eg., using endotoxin or lipopolysaccharide, LPS), have shown a tripartite of proinflammatory cytokines - interleukin-1β (IL-1β), IL-6 and tumor necrosis factor-alpha (TNFα) – to be the most significant and potent influences on a variety of neural, endocrine and behavioral measures (Dantzer et al., 2008). However, it is now evident that these and other cytokines are part of the brain’s neurochemical milieu operating independently of immune challenge, and which can be elicited by neurotransmitter and neuropeptide systems activated by psychogenic stressors (Albrecht et al., 2010; Spulber and Schultzberg, 2010; Perez Nievas et al., 2011; Stipursky et al., 2011; Wager-Smith and Markou, 2011; Barnum et al., 2012; Santello et al., 2012).

**FIGURE 3:**
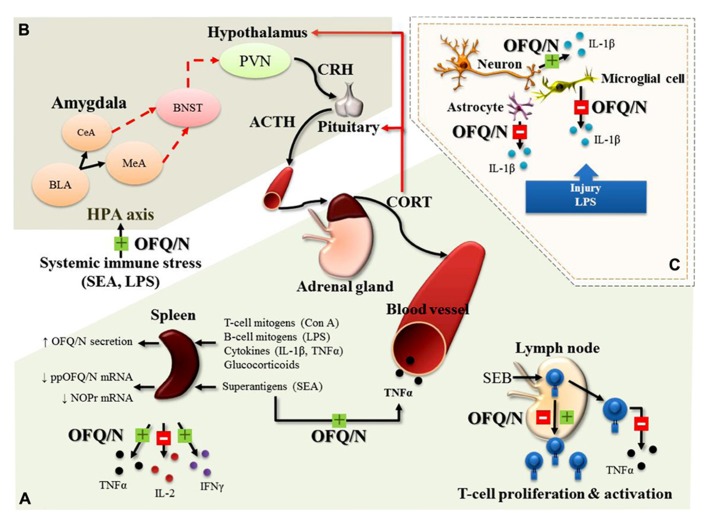
**Major, immunomodulatory effects of OFQ/N in the periphery (A)**, feed-forward effects of OFQ/N on the hypothalamic-pituitary-adrenal (HPA) axis response to systemic immune stress **(B)** and immunomodulatory effects of OFQ/N in the central nervous system (CNS; **C**). In general, the hypothesized role of the nociceptin system is believed to be anti-inflammatory, a function that may be exerted through regulation of cytokine expression. In response to a wide variety of immunologic stimuli (Con A, concanavalin A; LPS, lipopolysaccharide; IL-1β, Interleukin-1 beta; TNFα, tumor necrosis factor alpha; SEA, staphylococcal enterotoxin A) OFQ/N inhibits (red box with minus) or enhances (green box with plus) the release of inflammatory cytokines (TNFα, interleukin-2; IL-2 and interferon gamma; IFNγ) from splenocytes and lymphocytes and enhances SEA-induced elevations in circulating TNFα. Nociceptin also modulates T-lymphocyte proliferation and activation following superantigenic challenge with staphylococcal enterotoxin B (SEB; **A**). In line with its feed-forward effects on the HPA axis response to psychogenic stress, OFQ/N exerts stimulatory effects on the HPA axis response to systemic immune stress. Specifically, nociceptin signaling prolongs elevations in circulating CORT induced by systemic administration of SEA or LPS **(B)**. In the central nervous system (CNS), OFQ/N modulates the release inflammatory cytokines. Notably, nociceptin signaling appears to regulate, in a cell-specific manner, the expression of IL-1β. In response to traumatic injury or LPS, OFQ/N attenuates the release of IL-1β from microglial cells and astrocytes. Conversely, nociceptin signaling *enhances* the release of IL-1β from neurons **(C)**. Abbreviations used: corticotropin releasing hormone; CRH, adrenocorticotropin hormone; ACTH, paraventricular nucleus; PVN, bed nucleus of stria terminalis; BNST, central amygdala; CeA, medial amygdala; MeA, basolateral amygdala; BLA, basolateral amygdala.

An exciting development over the past decade is that OFQ/N is involved in regulation of neuroinflammatory and immunological processes. Orphanin FQ/nociceptin and its receptor are expressed in various peripheral tissues (Lachowicz et al., 1995; Mollereau et al., 1996) including immune cells and lymphoid organs (Wang et al., 1994), and similar to endogenous opioids, the OFQ/N system plays a role in immunomodulation (Halford et al., 1995; Kawashima et al., 2002; Kato et al., 2005; Rossi-George et al., 2005; Goldfarb et al., 2006; Miller and Fulford, 2007; Finley et al., 2008; Leggett et al., 2009b). Stimulation of NOP-R enhances or suppresses expression of proinflammatory cytokines (Buzas et al., 2002; Kawashima et al., 2002; Fu et al., 2006) or chemokines (Finley et al., 2008), stimulates chemotaxis (Serhan et al., 2001; Trombella et al., 2005), and modulates activation of the HPA axis in response to inflammatory stimuli (Leggett et al., 2009b; Mallimo et al., 2010). In general, the hypothesized role of the nociceptin system is believed to be anti-inflammatory, a function that may be exerted through regulation of cytokine expression. **Figure 3** provides a schematic illustration of the major findings discussed below, regarding the immunomodulatory role of OFQ/N in the periphery and CNS.

In the immune system, Miller and Fulford (2007) determined that OFQ/N secretion from lymphocytes and splenocytes (*in vitro*) is increased following exposure to various inflammatory mediators such as T (Concanavalin A; Con A) and B cell (LPS) mitogens, the proinflammatory cytokines IL-1β and TNFα, and synthetic glucocorticoids. Moreover, OFQ/N suppressed production of IL-2 from cultured splenocytes and significantly attenuated splenocyte proliferation in response to Con A stimulation, an effect that was inhibited by ORL-1 receptor antagonists (Miller and Fulford, 2007). There is also evidence to suggest that OFQ/N may have immunomodulatory properties *in vivo*. For instance, ORL-1^-/-^receptor deficient mice (ORL-1^-/-^) exhibited an exaggerated splenic IL-2 response 4 h after systemic challenge with the bacterial T lymphocyte “superantigen,” staphylococcal enterotoxin A (SEA; Mallimo et al., 2010). This finding is in good agreement with that of Miller and Fulford (2007) and the idea that signaling through the OFQ/N receptor limits the production of IL-2 following T cell activation.

On the other hand, intraperitoneal injection (ip) of OFQ/N to C57BL/6 mice, prior to challenge with SEA, produced an enhancement of splenic TNFα and IFNγ mRNA expression and increased plasma levels of TNFα (Goldfarb et al., 2006). Conversely, in the same study, ppOFQ/N^-/-^ mice challenged with SEA showed diminished mRNA for TNFα and IFNγ (ibid). The stimulatory effect of OFQ/N on TNFα and IFNγ may be necessary to ensure optimal levels of cytokine production at later time points following T cell stimulation. In line with this idea, ORL-1^-/-^ mice exhibited an attenuated splenic, TNFα response 4 h after systemic challenge with SEA (Mallimo et al., 2010). Collectively, these findings suggest that activation of the nociceptin system may play an important role in the balance of pro- and anti-inflammatory cytokine production. These effects of SEA may involve interactions at the level of T lymphocytes. That is, SEA is selective for T cells (Goldfarb et al., 2006), and T cells are known to express NOP-R, the receptor for OFQ/N (Peluso et al., 1998). Indeed, addition of OFQ/N to human T cell cultures stimulated with another superantigen, staphylococcal enterotoxin B (SEB), result in enhanced proliferation, but reduced TNFα production (Waits et al., 2004). Interestingly, restimulation of the same T cells resulted in OFQ/N causing a reduction in proliferative responding (ibid). A later study showed that cultured purified CD4+ T cells from human peripheral blood had attenuated proliferative responding to primary SEB exposure when OFQ/N was introduced into cell culture (Fiset et al., 2003; Easten et al., 2009). From these studies it is evident that OFQ/N has immunomodulatory properties that can target T cell function, although the direction of its effects on cytokine production and T cell proliferation require further characterization.

In keeping with *in vivo* studies that provided good evidence for OFQ/N-dependent immunomodulation (Goldfarb et al., 2006), additional studies are available to show the clinical applicability of these effects. In a rat study of experimental colitis, it was demonstrated that pathology and inflammatory cytokine production (eg., IL-1β) were diminished after two daily injections of low dose (0.02 and 0.2 nmol/Kg) OFQ/N, but were exacerbated when the dose was substantially increased (20 nmol/Kg; Petrella et al., 2013). The implications of this finding are consistent with an earlier observation that OFQ/N elevations may negatively impact survival during infection. That is, subsequent to the induction of sepsis by cecal ligation and puncture, OFQ/N administration to rats increased mortality, while NOP-R antagonist treatment attenuated various cellular and cytokine parameters of inflammation, and reduced mortality (Carvalho et al., 2008). Corresponding data in human clinical situations has been noted, with higher correlations of plasma OFQ/N in non-surviving septic patients suggesting that antagonism of OFQ/N may be a potential immunotherapeutic manipulation that may aid recovery (Serrano-Gomez et al., 2011). Overall, while little is known about the precise interactions between OFQ/N and the immune system, there is compelling evidence to explore this relationship further and elucidate the relevance of this to immunopathological and inflammatory disorders.

It is well established that systemic immune responses can produce changes in various neurobiological outcomes. In keeping with various CNS changes in neuropeptides and monamine neurotransmitters after immune challenge (Anisman et al., 2008), mice challenged with the bacterial T cell superantigen, SEA, showed increased ppOFQ/N mRNA in the hypothalamus and amygdala (Kawashima et al., 2002). In addition, SEA challenge activates the HPA axis and neuronal induction of the immediate early gene *c-fos* in various limbic brain regions, including the amygdala and hypothalamus (Rossi-George et al., 2005). Since these effects are known to depend on TNF (Rossi-George et al., 2005), it is possible that SEA-induced ppOFQ/N mRNA changes in the brain, are similarly dependent on the presence of TNF. Moreover, in the absence of ORL-1 signaling, the duration of the plasma CORT response to SEA (Mallimo et al., 2010) and to LPS (Leggett et al., 2009a) is reduced, suggesting that mobilization of central OFQ/N may promote a prolongation of the HPA axis response to systemic immune stress.

In addition to T cell antigens, strong proinflammatory inducers, such as LPS, which targets monocytes and macrophages, have been shown to induce OFQ/N production by cultured sensory neurons obtained from the dorsal root ganglion, an effect that appears to be dependent on the Toll-like receptor 4 (TLR4; Acosta and Davies, 2008). This may be relevant to nociception, as inflammation-related pain may be due to regulation of the hypoalgesic effects of OFQ/N, suggesting that NOP-R and OFQ/N are recruited by inflammatory stimuli to coordinate and control levels of pain perception (Depner et al., 2003; Chen and Sommer, 2007; Fu et al., 2007a). For instance, formalin and zymosan induce inflammation and induction of pain, although lower thresholds of pain sensitivity were evident in mice that lacked either ppOFQ/N and/or NOP-R (Depner et al., 2003). Furthermore, areas of inflammation in peripheral, as well as central, nervous tissue most likely attract infiltration of leukocytes (Prendergast and Anderton, 2009). Such cellular infiltrates may further contribute to the concentration of local neuron-derived OFQ/N, since neutrophils are known to produce OFQ/N (Fiset et al., 2003). Finally, as detailed below, astrocytes express NOP-R and cytokine production by astrocytes can be inhibited by OFQ/N, an effect which represents one mechanism by which OFQ/N may reduce pain sensitivity (Fu et al., 2007b).

In recent years there has been a resurgence of interest in glial cell function in the CNS, and in particular, how this contributes to and/or regulates neuroinflammatory processes in neurodegenerative conditions. The influence of OFQ/N on glial function has been suggested through a variety of different *in vitro* and *in vivo* studies. For example, NOP-R engagement may influence oligodendrocyte development, since the modulation of myelin basic protein concentrations subsequent to prenatal treatment with the opioid agonist, buprenorphine, were shown to be dependent on NOP-R expression (Eschenroeder et al., 2012). Similarly, with regard to astrocyte function, NOP-R knockout mice treated with methamphetamine have augmented GFAP expression in the striatum (Sakoori and Murphy, 2010), which is in keeping with other evidence that spinal cord astrocyte activation and *in vitro *cytokine production by astrocytes is attenuated by OFQ/N via ORL-1, which is expressed on astrocytes (Fu et al., 2007b). Interestingly, ppOFQ/N mRNA is increased in astrocytes treated with morphine (Takayama and Ueda, 2005), while LPS, and various proinflammatory cytokines (eg., tumor necrosis factor and interleukin-1) can increase astrocyte concentrations of immunoreactive OFQ/N and ppOFQ/N mRNA (Buzas et al., 2002). Finally, following neural trauma in rats, LPS treatment increased OFQ/N co-localized with brain astrocytes (Zhao et al., 2002). This observation was made in the context of a corresponding investigation of microglial cells, and is perhaps the only major instance where OFQ/N was linked to this intensively studied category of glial cells.

Microglia essentially are the tissue macrophages of the brain and spinal cord, and as such, likely exert an “immune-like” influence through phagocytosis and production of proinflammatory cytokines like TNFα and IL-1β. In the study by Zhao et al. (2002), rats given icv infusion of OFQ/N showed a significantly reduced number of activated microglia following neural trauma. In addition, LPS induced upregulation of IL-1β mRNA in cultured microglial cells, and this was inhibited by application of OFQ/N (Fu et al., 2006). This effect was blocked by co-administration of an ORL-1 receptor antagonist, confirming that downregulation of Il-1β mRNA transcripts in microglia of the CNS is ORL-1 dependent. However, when applied to cultured neurons, OFQ/N significantly increased LPS-induced upregulation of IL-1β mRNA (Fu et al., 2006). The differential effect of OFQ/N on IL-1β gene expression on microglial and neuronal preparations was suggested to reflect a disturbance in the endogenous synaptic structural arrangement between these cell types. Thus, microglia *in vivo* may exert a direct inhibitory action on neurons and when this connection is disturbed, neurons may upregulate IL-1β mRNA instead (Fu et al., 2006). This is a compelling hypothesis, that is hampered by a relative paucity of additional studies to corroborate and expand on the effects of OFQ/N on microglial cells.

In conclusion, although relatively small, there is a potentially significant literature relating OFQ/N to glial cell function. A compelling view that might be elaborated and pursued is that of OFQ/N mediated reduction of proinflammatory cytokine production during various conditions of trauma, degeneration and systemic inflammation due to infection and other immunologically relevant conditions (eg., autoimmune disease). It seems plausible, for example, that OFQ/N may also serve to modify the various sickness behaviors – cognitive, motoric and emotional – that arise from exposure to immunological stimuli. This would fit the overall – but not overwhelming – view that OFQ/N serves to attenuate stress-related behavioral reactions, as reviewed earlier. Extending this idea further, it has been demonstrated that stress can activate microglial cells in the brain (Sugama et al., 2007, 2009; Hinwood et al., 2012), and furthermore, stress can also increase the number of astrocytes that express IL-1β (Sugama et al., 2011). Given that psychogenic and immunologic stressors can increase OFQ/N expression in the brain (Kawashima et al., 2002; Nativio et al., 2012), it could be hypothesized that an increase in OFQ/N may serve to regulate glial cell activation and the production of cytokines, like IL-1β (Zhao et al., 2002). Whether such interactions exist between central OFQ/N changes and neuroinflammatory events remains to be determined.

## SUMMARY AND FUTURE DIRECTIONS

The current review has focused on bringing together three elements of OFQ/N biology that are potentially linked, and may hold promise for future studies on brain health and neuroplasticity. Given that many of the studies addressing the role of OFQ/N on the HPA axis and anxiety-related behavior have focused on acute stressor exposure, greater attention is needed to address the relationship of OFQ/N to neuroplasticity during conditions of chronic stress. Chronic stress has long been associated as a contributor and/or trigger for anxiety and mood disorders(Charney, 2003; Feder et al., 2009), and the pursuit of the molecular and cellular targets of stress-activated neurotransmitter systems (eg., the monoamines) has shifted to the importance of neuroinflammatory cytokines and glial cells, which express receptors for multiple neurotransmitters (Kettenmann et al., 2011). Indeed, as stated above, microglial cells, are activated under a range of conditions, including strokes, physical trauma, demyelination, and neurodegeneration or neuronal cell death (Ransohoff and Perry, 2009). Of particular interest is that the activated phenotype of a microglial cell can be produced by *in vitro* exposure to neurotransmitters (eg., norepinephrine; Heneka et al., 2010), as well as psychogenic stressors, which increase neurotransmitter synthesis and release(Anisman et al., 2005). Indeed, psychogenic stressors alone, in the absence of immune activation in the periphery, can activate brain microglial cells and modify cytokine levels in the brain (Frank et al., 2007; Wohleb et al., 2011; Hinwood et al., 2012; Gibb et al., 2008, 2011). What is relevant and novel to this scenario is the knowledge that OFQ/N plays an immunomodulatory role that can modify a number of proinflammatory cytokines, including IL-1β, TNF-α and IL-2. This serves to move the relationship of OFQ/N to stress and behavior, into a scheme that also incorporates the contribution of neuroinflammatory functions that might be regulated or modified by OFQ/N. This new direction may yield clues for the treatment of neuropsychiatric clinical conditions, in which neuroinflammation is suspected of contributing to depression, cognitive decline and neurodegeneration.

## Conflict of Interest Statement

The authors declare that the research was conducted in the absence of any commercial or financial relationships that could be construed as a potential conflict of interest.
